# Supplementary material for: Evaluating the fitness of PA/I38T-substituted influenza A viruses with reduced baloxavir susceptibility in a competitive mixtures ferret model
Source: PLoS Pathog. 2021 May 6;17(5):e1009527. doi: 10.1371/journal.ppat.1009527 (PMC8130947; doi:10.1371/journal.ppat.1009527)
Supplement: S1 Fig — MDCK cells were co-infected with reverse genetics-derived WT and PA/I38-substitutited viruses at 50:50 ratio based on viral titers at an MOI of 0.001 each. At 48 hours post infection, the culture supernatant was collected and serially passaged three times. The virus passage experiments were conducted in duplicate as lineage 1 and lineage 2. Each culture supernatant was subjected to Sanger sequencing to analyze the change of amino acid at 38 in PA subunit. Sanger sequence chromatograms of amino acid at position 38 in the PA subunit were analyzed using BioPython, and blue chromatograph represents WT and red shows PA/I38-substituted viruses. Representative sequencing chromatographs of lineage 1 were shown because the similar results were obtained from both lineages. P0–P3, passage 0 to passage 3. (DOCX) [file ppat.1009527.s001.docx]

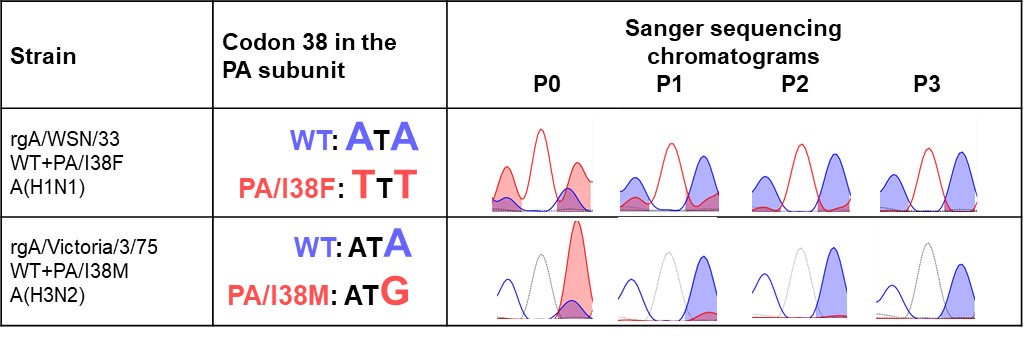


**S1 Fig. Competitive replicative capacity of influenza reverse genetics-derived viruses with PA/I38 substitutions**
