# Supplementary material for: Evaluating the fitness of PA/I38T-substituted influenza A viruses with reduced baloxavir susceptibility in a competitive mixtures ferret model
Source: PLoS Pathog. 2021 May 6;17(5):e1009527. doi: 10.1371/journal.ppat.1009527 (PMC8130947; doi:10.1371/journal.ppat.1009527)
Supplement: S2 Fig — MDCK cells were co-infected with reverse genetics-derived WT and PA/I38T-substituted viruses at 50:50 ratios based on viral titers at a MOI of 0.001 each, in the presence of various concentrations of baloxavir. EC50 values were 0.42 nmol/L and 1.13 nmol/L for rgA/WSN/33 (H1N1) and rgA/Victoria/3/75 (H3N2) viruses, respectively, based on plaque reduction assay results previously reported [15]. At 48 hours post infection, the culture supernatant was collected and serially passaged three times. The virus passage experiments were conducted in duplicate as lineage 1 and lineage 2. Each culture supernatant was subjected to Sanger sequencing to analyze the change of amino acid at position 38 in PA subunit. Sanger sequence chromatograms of amino acid at position 38 in the PA subunit were analyzed using BioPython; the blue chromatograph represents WT and the red shows PA/I38T-substituted viruses. Representative sequencing chromatographs of lineage 1 were shown because similar results were obtained from both lineages. BXA, baloxavir acid; MOI, multiplicity of infection; N.T., not tested; P0–P3, passage 0 to passage 3. Low virus titer indicates where viruses were not passaged due to a low virus titer. (DOCX) [file ppat.1009527.s002.docx]

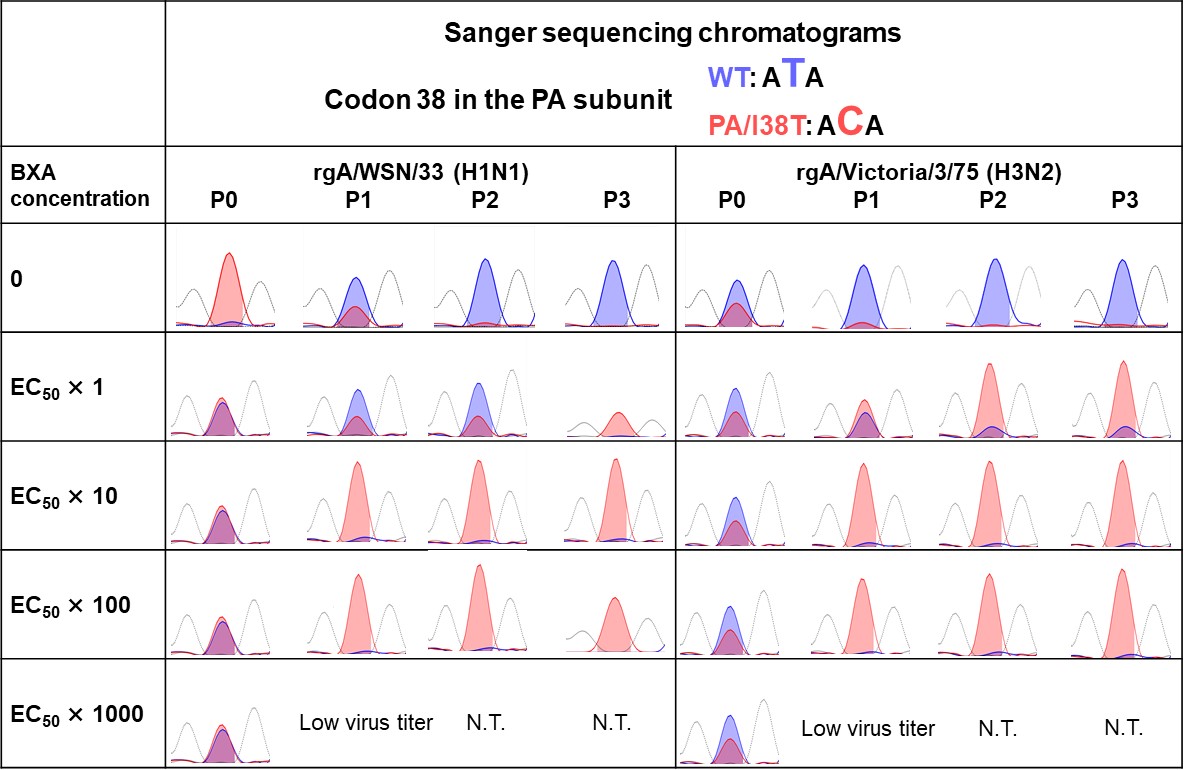


**S2 Fig. Competitive replicative capacity of influenza reverse genetics-derived viruses with PA/I38T substitutions in the presence of baloxavir**
