# Supplementary material for: Evaluating the fitness of PA/I38T-substituted influenza A viruses with reduced baloxavir susceptibility in a competitive mixtures ferret model
Source: PLoS Pathog. 2021 May 6;17(5):e1009527. doi: 10.1371/journal.ppat.1009527 (PMC8130947; doi:10.1371/journal.ppat.1009527)
Supplement: S3 Fig — Flow diagram of sample selection for fitness study in vitro and ferret transmission study for (A) influenza A/H1N1pdm09 and (B) influenza A/H3N2 viruses. Polymorphic amino acid substitutions in PA protein aligned with (A) influenza A/California/7/2009 (A/H1N1) and (B) influenza A/Texas/50/2012 (A/H3N2) are also shown. (DOCX) [file ppat.1009527.s003.docx]

**Supplementary figures**


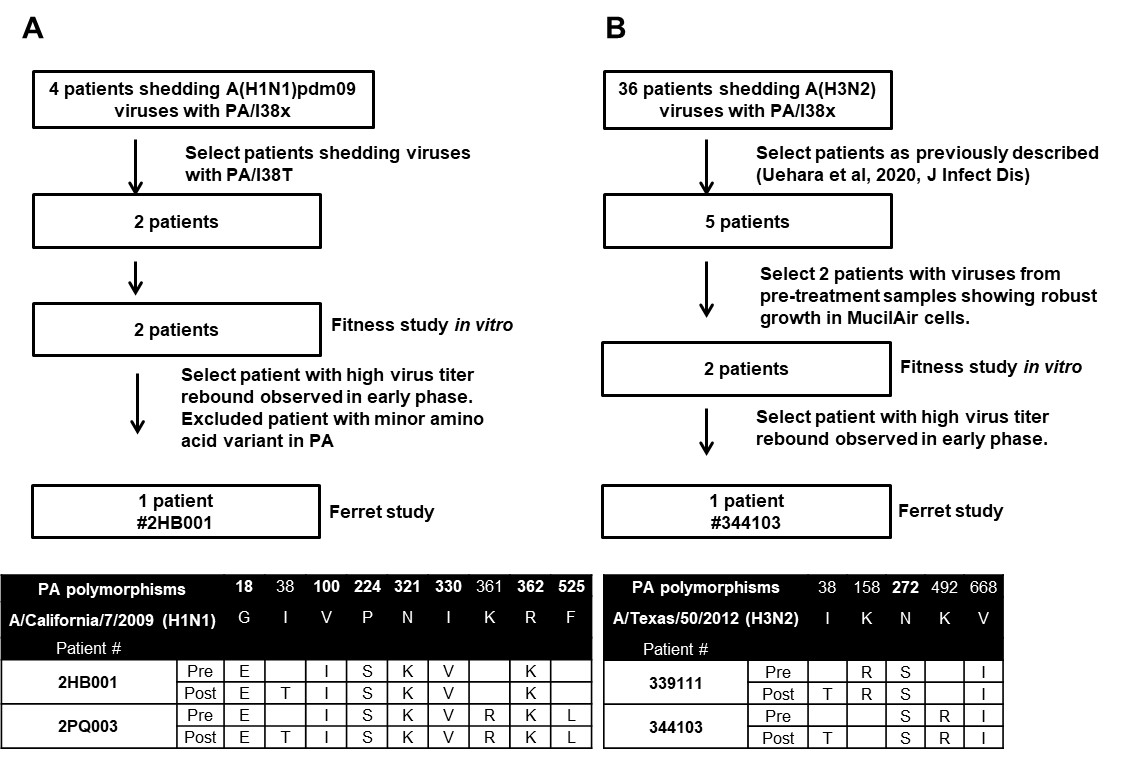


**S3 Fig. Sample selection for fitness study *in vitro* and ferret transmission study**
