# Supplementary figures and images for: Evaluating the fitness of PA/I38T-substituted influenza A viruses with reduced baloxavir susceptibility in a competitive mixtures ferret model
Source: PLoS Pathog. 2021 May 6;17(5):e1009527. doi: 10.1371/journal.ppat.1009527 (PMC8130947; doi:10.1371/journal.ppat.1009527)

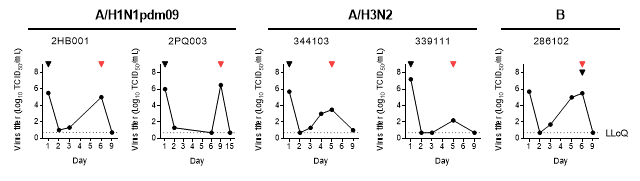


**S4 Fig. Influenza virus titer (log10 TCID50/mL) in individual patients with PA/I38T-substituted viruses**

Supplement: S4 Fig — Time course of influenza virus titer of individual baloxavir-treated patients with PA/I38X-substituted viruses, determined by TCID50 assay (values below LLOQ were set at 0.7 log10 TCID50/mL). The black and red arrowheads indicate the sampling time-points for WT viruses (pre-baloxavir treatment for type A viruses and post-baloxavir treatment for type B virus) and PA/I38T-substituted viruses (post-baloxavir treatment), respectively. Black dotted line = LLOQ at 0.7 log10 TCID50. (DOCX) [file ppat.1009527.s004.docx]

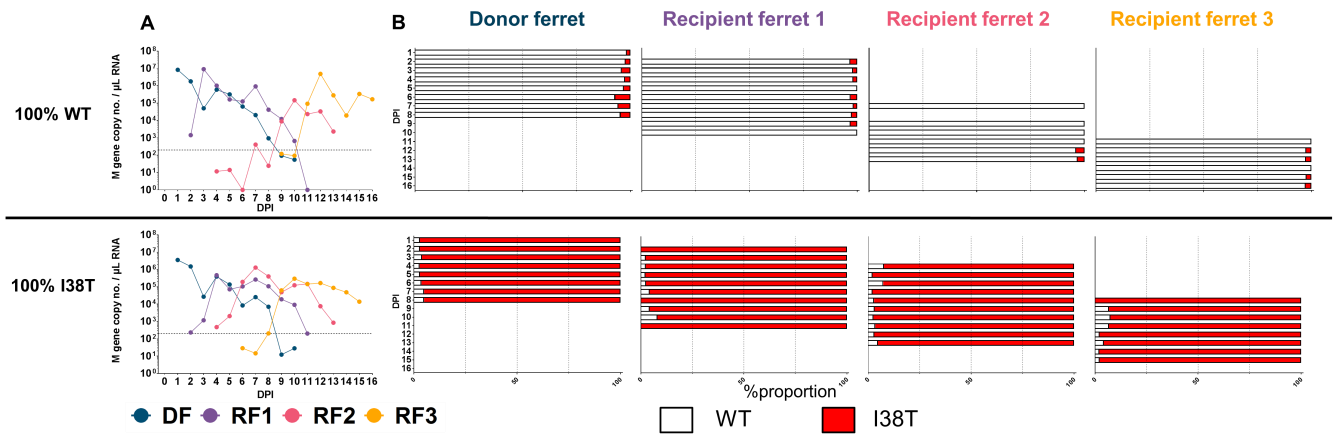


**S5 Fig. Pyrosequencing of ferret nasal washes from WT or PA/I38T-variant A/H3N2 pure population groups**

Supplement: S5 Fig — (A) Viral RNA and (B) pyrosequencing of ferret nasal washes from A/H3N2 WT or PA/I38T pure population groups. (DOCX) [file ppat.1009527.s005.docx]
