# Supplementary material for: Evaluating the fitness of PA/I38T-substituted influenza A viruses with reduced baloxavir susceptibility in a competitive mixtures ferret model
Source: PLoS Pathog. 2021 May 6;17(5):e1009527. doi: 10.1371/journal.ppat.1009527 (PMC8130947; doi:10.1371/journal.ppat.1009527)
Supplement: S6 Fig — (A) Viral RNA and (B) pyrosequencing of ferret nasal washes from A/H1N1pdm09 WT or PA/I38T pure population groups. (DOCX) [file ppat.1009527.s006.docx]

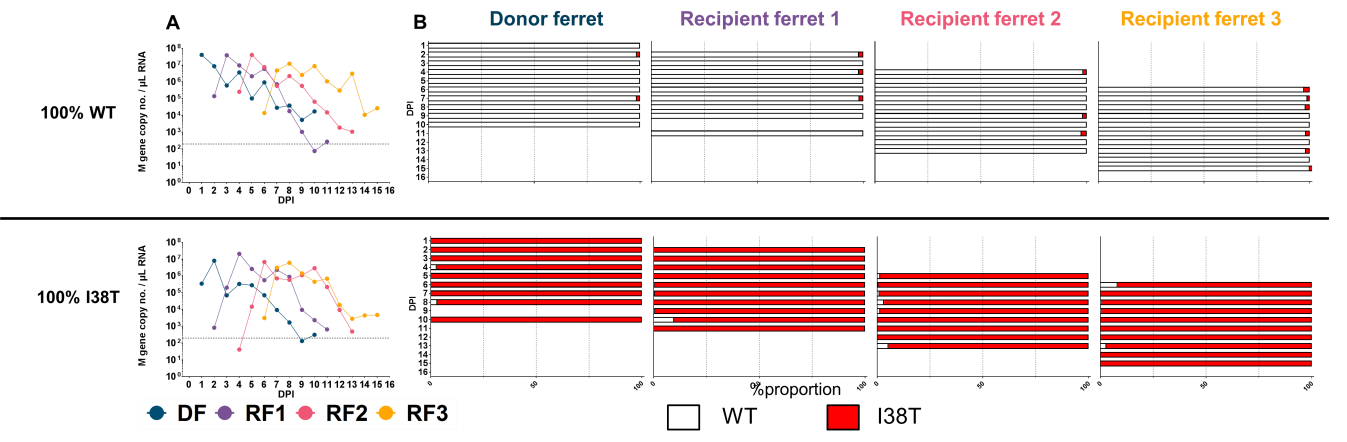


**S6 Fig. Pyrosequencing of ferret nasal washes from WT or PA/I38T-variant A/H1N1pdm09 pure population groups**
