# Supplementary material for: Evaluating the fitness of PA/I38T-substituted influenza A viruses with reduced baloxavir susceptibility in a competitive mixtures ferret model
Source: PLoS Pathog. 2021 May 6;17(5):e1009527. doi: 10.1371/journal.ppat.1009527 (PMC8130947; doi:10.1371/journal.ppat.1009527)
Supplement: S7 Fig — (DOCX) [file ppat.1009527.s007.docx]

| **Type/subtype** | **Patient ID** | **Ratio of WT to PA/I38T** | **Titer (TCID_50_/mL)** | |
| --- | --- | --- | --- | --- |
|  |  |  | P1 | P2 |
| A/H1N1pdm09 | 2HB001 | 50:50 | 4.64E+07 | 4.64E+07 |
|  |  |  | 4.64E+06 | 3.73E+07 |
|  |  | 20:80 | 6.31E+07 | 1.00E+07 |
|  |  |  | 1.58E+07 | 3.16E+08 |
|  | 2PQ003 | 50:50 | 4.64E+06 | 1.95E+07 |
|  |  |  | 7.94E+06 | 1.95E+07 |
|  |  | 20:80 | 1.00E+06 | 1.31E+08 |
|  |  |  | 6.31E+05 | 4.64E+07 |
| A/H3N2 | 339111 | 50:50 | 6.31E+05 | 6.31E+05 |
|  |  |  | 6.31E+04 | 2.15E+06 |
|  |  | 20:80 | 7.91E+05 | 2.15E+07 |
|  |  |  | 1.58E+06 | 1.95E+07 |
|  | 344103 | 50:50 | 3.73E+04 | 5.62E+05 |
|  |  |  | 3.89E+06 | 4.64E+05 |
|  |  | 20:80 | 7.91E+05 | 2.68E+06 |
|  |  |  | 6.42E+06 | 3.73E+06 |
| B | 286102 | 50:50 | 4.64E+07 | 2.68E+08 |
|  |  |  | 6.31E+07 | 1.00E+08 |
|  |  | 20:80 | 2.15E+07 | 3.89E+07 |
|  |  |  | 4.64E+07 | 3.16E+07 |

**S7 Fig. Infectious viral titers (TCID_50_) of MucilAir cell supernatant used for serial passaging in manuscript Figure 1**
