# Supplementary material for: Evaluating the fitness of PA/I38T-substituted influenza A viruses with reduced baloxavir susceptibility in a competitive mixtures ferret model
Source: PLoS Pathog. 2021 May 6;17(5):e1009527. doi: 10.1371/journal.ppat.1009527 (PMC8130947; doi:10.1371/journal.ppat.1009527)
Supplement: S8 Fig — (A) A/H3N2 viral titers (B). A/H1N1pdm09 viral titers. (DOCX) [file ppat.1009527.s008.docx]

**A.** Clinical A/H3N2

**B.** Clinical A/H1N1pdm09

**S8 Fig. Influenza virus titer (log_10_TCID_50_/mL) of ferret nasal washes from competitive mixture direct contact transmission chains (Melbourne)**
