# Supplementary material for: Evaluating the fitness of PA/I38T-substituted influenza A viruses with reduced baloxavir susceptibility in a competitive mixtures ferret model
Source: PLoS Pathog. 2021 May 6;17(5):e1009527. doi: 10.1371/journal.ppat.1009527 (PMC8130947; doi:10.1371/journal.ppat.1009527)
Supplement: S9 Fig — (DOCX) [file ppat.1009527.s009.docx]

**
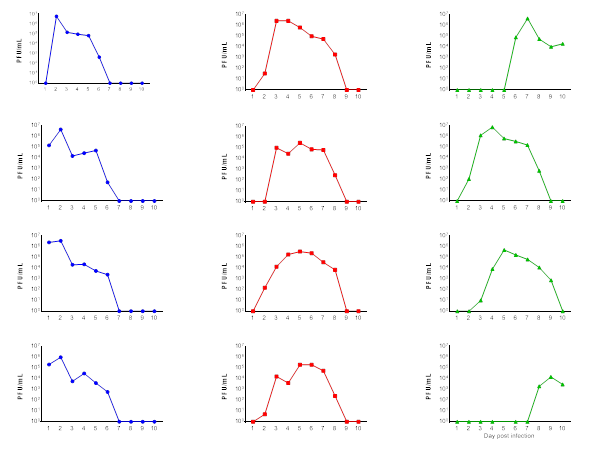
S9 Fig. Influenza virus titer (PFU/mL) of ferret nasal washes from transmission of recombinant A/H1N1pdm09 competition experiments (London)**
