# Supplementary material for: Evaluating the fitness of PA/I38T-substituted influenza A viruses with reduced baloxavir susceptibility in a competitive mixtures ferret model
Source: PLoS Pathog. 2021 May 6;17(5):e1009527. doi: 10.1371/journal.ppat.1009527 (PMC8130947; doi:10.1371/journal.ppat.1009527)
Supplement: S1 Table — (DOCX) [file ppat.1009527.s010.docx]

**S1 Table.** Overview of synonymous and non-synonymous mutations with ≥5% frequency in ferret nasal washes of PA/I38T-infected ferrets

| **Gene** | **Mutation** | **Frequency** | **Synonymous?** |
| --- | --- | --- | --- |
| **A/H1N1, 100% PA/I38T RF3 (Melbourne)** | | | |
| PB1 | G1679A | 7% | No (R560K) |
| **A/H3N2, 100% PA/I38T RF3 (Melbourne)** | | | |
| HA | C885T | 100% | Yes |
| NP | G1302A | 30% | Yes |
| PA | G78A | 7% | Yes |
| PB2 | G265A | 68% | No (V89M) |
| **A/H3N2, 20% WT : 80% PA/I38T RF3 (Melbourne)** | | | |
| HA | C950T | 51% | No (T317I) |
| PB1 | G1095A | 47% | Yes |
| **A/H1N1, PA/I38T (London, ferret 408)** | | | |
| PA | C513A (Day 1)  T696C (Day 1)  G126A (Day 4)  G126A (Day 5) | 5%  7%  8%  8% | Yes  Yes  Yes  Yes |
| HA | A424G  Day 1  Day 3  Day 4  Day 5  Day 6  A716G (Day 5)  A772G (Day 5)  G1117A (Day 1)  G1497T  Day 1  Day 2  Day 3  Day 4  Day 5  Day 6  Day 7 | 9%  9%  9%  6%  5%  7%  9%  15%  33%  24%  29%  29%  26%  15%  16% | No (N142D)  No (N142D)  No (N142D)  No (N142D)  No (N142D)  No (D239G)  Yes  No (E373K)  Yes  Yes  Yes  Yes  Yes  Yes  Yes |
| NA | C854T (Day 3) | 6% | No (S285F) |
| NP | G864C (Day 7) | 29% | Yes |
| NS | T751C (Day 3) | 5% | Yes |
| PB1 | C1005T (Day 3)  C1005T (Day 4)  A386G (Day 5)  G1710A (Day 6) | 7%  5%  8%  28% | Yes  Yes  No (Y129C)  Yes |
| PB2 | G2199A (Day 2)  T274C (Day 5) | 9%  9% | Yes  No (S92P) |
| **A/H1N1, PA/I38T (London, ferret 409)** | | | |
| HA | A315G (Day 2)  G721A (Day 7)  G1117A (Day 6)  G1497T  Day 2  Day 3  Day 4  Day 5  Day 6  Day 7  Day 8  Day 9 | 5%  14%  8%  30%  24%  18%  18%  7%  24%  24%  21% | Yes  No (E241K)  No (E373K)  Yes  Yes  Yes  Yes  Yes  Yes  Yes  Yes |
| NA | G247T (Day 2) | 6% | No (V83L) |
| PA | G372A (Day 2)  C559T (Day 2)  G1074A (Day 2) | 8%  6%  6% | Yes  Yes  Yes |
| PB1 | C462T (Day 2)  G1017A (Day 2)  C1202T (Day 7)  C1446T (Day 2)  A1968C  Day 6  Day 7  T2126C (Day 4) | 6%  9%  36%  7%  10%  9%  9% | Yes  Yes  No (A401V)  Yes  Yes  Yes  No (V709A) |
| PB2 | A751G (Day 2)  G1750A (Day 2)  A1659G  Day 3  Day 4  Day 5  Day 6  Day 7  Day 8  Day 9 | 6%  6%  7%  15%  51%  21%  30%  19%  33% | No (R251G)  No (V584I)  Yes  Yes  Yes  Yes  Yes  Yes  Yes |
| **A/H1N1, PA/I38T (London, ferret 410)** | | | |
| HA | A424G  Day 6  Day 7  Day 8  Day 9  Day 10  G721A (Day 10)  G1497T  Day 6  Day 7  Day 8  Day 9  Day 10 | 100%  100%  100%  100%  100%  24%  100%  100%  100%  100%  100% | No (N142D)  No (N142D)  No (N142D)  No (N142D)  No (N142D)  No (E241K)  Yes  Yes  Yes  Yes  Yes |
| NS | G739A (Day 10) | 43% | Yes |
| PB1 | C125G  Day 7  Day 8  Day 9  Day 10 | 14%  5%  18%  18% | No (T42R)  No (T42R)  No (T42R)  No (T42R) |
| PB2 | A80G  Day 7  Day 9  Day 10  G1156A  Day 6  Day 7  Day 8  Day 9  Day 10 | 5%  14%  7%  100%  100%  100%  100%  100% | No (H27R)  No (H27R)  No (H27R)  No (V386I)  No (V386I)  No (V386I)  No (V386I)  No (V386I) |
| **A/H1N1, PA/I38T (London, ferret 411)** | | | |
| HA | A424G  Day 1  Day 2  Day 3  Day 4  Day 6  Day 7  G1497T  Day 1  Day 2  Day 3  Day 4  Day 5  Day 6  Day 7 | 18%  8%  7%  14%  8%  18%  39%  26%  31%  33%  25%  20%  43% | No (N142D)  No (N142D)  No (N142D)  No (N142D)  No (N142D)  No (N142D)  Yes  Yes  Yes  Yes  Yes  Yes  Yes |
| PA | G173C  Day 5  Day 6  Day 7 | 12%  7%  12% | No (G58A)  No (G58A)  No (G58A) |
| PB2 | G412A (Day 6)  A1522G | 14%  53% | No (E72K)  No (R508G) |
| **A/H1N1, PA/I38T (London, ferret 412)** | | | |
| HA | A424G  Day 4  Day 5  Day 6  Day 7  Day 8  Day 9  G721A  Day 7  Day 8  A820G  Day 6  Day 7  Day 8  G1117A (Day 5)  G1146A (Day 6)  G1497T  Day 2  Day 4  Day 5  Day 6  Day 7  Day 8  Day 9 | 28%  45%  29%  36%  38%  43%  7%  14%  6%  21%  14%  12%  12%  15%  37%  40%  27%  29%  49%  50% | No (N142D)  No (N142D)  No (N142D)  No (N142D)  No (N142D)  No (N142D)  No (E241K)  No (E241K)  No (M274V)  No (M274V)  No (M274V)  No (E373K)  Yes  Yes  Yes  Yes  Yes  Yes  Yes  Yes |
| PA | G126A  Day 3  Day 4  Day 6  Day 7  Day 8 | 26%  10%  6%  12%  8% | Yes  Yes  Yes  Yes  Yes |
| PB1 | C60T  Day 3  Day 4  Day 6  Day 7  Day 8  A1122G  Day 6  Day 7  G1966C  Day 5  Day 8 | 23%  24%  7%  7%  17%  32%  32%  8%  26% | Yes  Yes  Yes  Yes  Yes  Yes  Yes  No (E656Q)  No (E656Q) |
| PB2 | T106C  Day 3  Day 4  A196C  Day 5  Day 6  G214A  Day 4  Day 5  Day 6  Day 7  Day 8  G1156A  Day 4  Day 5  Day 6  Day 7  Day 8  T1518A (Day 5)  A1522G (Day 5)  A2109G  Day 3  Day 4  Day 5  Day 6  Day 7  A2136C (Day 6) | 7%  22%  13%  7%  21%  22%  7%  13%  44%  24%  40%  28%  17%  42%  22%  54%  10%  84%  5%  12%  10%  6% | S36P  S36P  No (M66L)  No (M66L)  No (E72K)  No (E72K)  No (E72K)  No (E72K)  No (E72K)  No (V386I)  No (V386I)  No (V386I)  No (V386I)  No (V386I)  Yes  No (R508G)  Yes  Yes  Yes  Yes  Yes  Yes |
| **A/H1N1, PA/I38T (London, ferret 413)** | | | |
| HA | A424G  Day 2  Day 3  Day 4  Day 5  Day 6  Day 7  Day 8  Day 9  G721A  Day 7  Day 8  G1497T  Day 2  Day 3  Day 4  Day 5  Day 6  Day 7  Day 8  Day 9 | 94%  100%  100%  100%  100%  84%  95%  >95%  14%  27%  99%  100%  100%  100%  100%  100%  100%  100% | No (N142D)  No (N142D)  No (N142D)  No (N142D)  No (N142D)  No (N142D)  No (N142D)  No (N142D)  No (E241K)  No (E241K)  Yes  Yes  Yes  Yes  Yes  Yes  Yes  Yes |
| PA | C1601A (Day 2)  C1603T  Day 2  Day 3  Day 4  Day 5  Day 6  Day 7  Day 8  Day 9 | 30%  83%  100%  100%  100%  100%  100%  93%  17% | No (P534Q)  No (H535Y)  No (H535Y)  No (H535Y)  No (H535Y)  No (H535Y)  No (H535Y)  No (H535Y)  No (H535Y) |
| PB2 | G2077A (Day 7) | 7% | No (G693R) |
| **A/H1N1, PA/I38T (London, ferret 414)** | | | |
| HA | A424G  Day 1  Day 7  G1497T  Day 1  Day 2  Day 3  Day 4  Day 5  Day 6  Day 7 | 7%  6%  24%  29%  27%  36%  45%  38%  53% | No (N142D)  No (N142D)  Yes  Yes  Yes  Yes  Yes  Yes  Yes |
| NA | A658G | 21% | R220G |
| NS | G537T  Day 6  Day 7 | 21%  16% | Yes  Yes |
| PB1 | A1968C | 5% | Yes |
| PB2 | T1518A  Day 5  Day 6  T1518A  Day 5  Day 6 | 10%  25%  35%  67% | Yes  Yes  No (R508G)  No (R508G) |
| **A/H1N1, PA/I38T (London, ferret 415)** | | | |
| HA | G721A  Day 7  Day 8  G1012A  Day 4  Day 5  Day 6  Day 9  Day 10  G1497T  Day 2  Day 3  Day 4  Day 5  Day 6  Day 7  Day 8  Day 9  Day 10 | 9%  19%  11%  39%  20%  6%  8%  16%  >95%  82%  27%  30%  74%  54%  61%  75% | No (E241K)  No (E241K)  No (V338I)  No (V338I)  No (V338I)  No (V338I)  No (V338I)  Yes  Yes  Yes  Yes  Yes  Yes  Yes  Yes  Yes |
| NP | G77A  Day 4  Day 5  Day 6  Day 7  Day 8  Day 9 | 15%  44%  24%  11%  23%  11% | No (R26K)  No (R26K)  No (R26K)  No (R26K)  No (R26K)  No (R26K) |
| M | T771G  Day 5  Day 6  Day 7  Day 8  Day 9  Day 10 | 17%  17%  7%  8%  17%  24% | Yes  Yes  Yes  Yes  Yes  Yes |
| NS | A633G  Day 5  Day 6 | 6%  6% | Yes  Yes |
| PB2 | C41T  Day 3  Day 4  Day 5  Day 6  Day 7  Day 8  Day 9  A708G (Day 2)  T1006C (Day 2)  A1792G  Day 4  Day 5  Day 6  Day 8 | 51%  30%  21%  22%  31%  8%  15%  22%  28%  11%  33%  17%  25% | No (S14F)  No (S14F)  No (S14F)  No (S14F)  No (S14F)  No (S14F)  No (S14F)  Yes  No (S336P)  No (T598A)  No (T598A)  No (T598A)  No (T598A) |
| **A/H1N1, PA/I38T (London, ferret 416)** | | | |
| HA | A722G (Day 8)  G1497T  Day 4  Day 5  Day 6  Day 7  Day 8  Day 9  Day 10 | 10%  21%  15%  24%  30%  31%  25%  22% | No (E241G)  Yes  Yes  Yes  Yes  Yes  Yes  Yes |
| PA | G238A  Day 6  Day 7  Day 8  Day 9  A472G  Day 4  Day 5  Day 6  Day 7  Day 8  Day 9  A1947T  Day 7  Day 8 | 9%  13%  16%  5%  78%  63%  48%  60%  32%  60%  6%  6% | No (E80K)  No (E80K)  No (E80K)  No (E80K)  No (K158E)  No (K158E)  No (K158E)  No (K158E)  No (K158E)  No (K158E)  Yes  Yes |
| PB1 | A216G  Day 4  Day 5  Day 6  Day 7  Day 8  Day 9 | 6%  14%  12%  5%  19%  7% | Yes  Yes  Yes  Yes  Yes  Yes |
| PB2 | C166A  Day 6  Day 7  Day 8  Day 9  T1994A  Day 5  Day 6  Day 7  Day 8  Day 9 | 10%  5%  15%  7%  8%  9%  7%  30%  16% | No (P56T)  No (P56T)  No (P56T)  No (P56T)  No (L665H)  No (L665H)  No (L665H)  No (L665H)  No (L665H) |
| **A/H1N1, PA/I38T (London, ferret 417)** | | | |
| HA | C405T  Day 5  Day 3  A424G  Day 2  Day 3  Day 5  G1497T  Day 2  Day 3  Day 4  Day 5  Day 6  Day 7  Day 8 | 15%  11%  7%  5%  6%  33%  36%  34%  41%  33%  32%  72% | Yes  Yes  No (N142D)  No (N142D)  No (N142D)  Yes  Yes  Yes  Yes  Yes  Yes  Yes |
| PB2 | G633A (Day 6) | 22% | Yes |
| **A/H1N1, PA/I38T (London, ferret 418)** | | | |
| HA | A424G  Day 3  Day 4  Day 5  Day 6  Day 7  Day 8  Day 9  Day 10  G721A  Day 7  Day 8  C1143T  Day 5  Day 6  Day 7  Day 8  Day 9  Day 10  G1173A  Day 4  Day 5  Day 6  Day 7  Day 8  Day 9  Day 10  G1497T  Day 3  Day 4  Day 5  Day 6  Day 7  Day 8  Day 9  Day 10 | 7%  36%  51%  23%  41%  24%  38%  23%  14%  12%  7%  12%  6%  8%  24%  16%  27%  29%  16%  26%  25%  25%  19%  37%  39%  49%  34%  50%  51%  63%  48% | No (N142D)  No (N142D)  No (N142D)  No (N142D)  No (N142D)  No (N142D)  No (N142D)  No (N142D)  No (E241K)  No (E241K)  Yes  Yes  Yes  Yes  Yes  Yes  Yes  Yes  Yes  Yes  Yes  Yes  Yes  Yes  Yes  Yes  Yes  Yes  Yes  Yes  Yes |
| NA | C300T (Day 4) | 10% | Yes |
| PA | T124A  Day 4  Day 7  Day 8  T1257A  Day 4  Day 5  Day 6  Day 7  Day 8  Day 9  Day 10  T1946C  Day 7  Day 8 | 7%  7%  9%  35%  57%  57%  53%  59%  71%  52%  6%  12% | No (L42M)  No (L42M)  No (L42M)  Yes  Yes  Yes  Yes  Yes  Yes  Yes  No (L649P)  No (L649P) |
| NS | G223A  Day 4  Day 5  Day 6  Day 7  Day 8  Day 9  Day 10  A525C  Day 5  Day 6  Day 7  Day 8  Day 10 | 8%  18%  12%  19%  19%  18%  22%  11%  14%  11%  6%  6% | No (E75K)  No (E75K)  No (E75K)  No (E75K)  No (E75K)  No (E75K)  No (E75K) |
| M | C249T  Day 4  Day 7  Day 8  C519T  Day 5  Day 6  Day 9  Day 10 | 9%  5%  7%  6%  12%  6%  8% |  |
| PB1 | C1023A  Day 5  Day 6  Day 7  Day 8  Day 10  G1587A (Day 4) | 10%  5%  9%  10%  14%  11% | Yes  Yes  Yes  Yes  Yes  Yes |
| PB2 | T197A  Day 4  Day 5  Day 7  Day 8  Day 10 | 18%  9%  9%  16%  7% | No (M66K)  No (M66K)  No (M66K)  No (M66K)  No (M66K) |
| **A/H1N1, PA/I38T (London, ferret 419)** | | | |
| HA | G1497T  Day 8  Day 9  Day 10 | 100%  100%  100% | Yes  Yes  Yes |
| NS | A387G  Day 9  Day 10 | 13%  13% | Yes  Yes |
| PB1 | G243A  Day 8  Day 9  Day 10 | 27%  49%  17% | Yes  Yes  Yes |
| **A/H1N1, PA/I38T (London, ferret 356)** | | | |
| HA | G408T (Day 3)  T698A (Day 3)  G1366T (Day 3) | 29%  100%  21% | Yes  No (I233K)  No (D456Y) |
| PB1 | T261G (Day 7) | 38% | Yes |
| PB2 | T318A (Day 3) | 100% | Yes |
| **A/H1N1, PA/I38T (London, ferret 359)** | | | |
| HA | A697G  Day 2  Day 4  T698A  Day 2  Day 4  G1366T  Day 2  Day 4 | 54%  32%  100%  100%  83%  57% | No (K233V)  No (K233V)  No (I233K)  No (I233K)  No (D456Y)  No (D456Y) |
| PA | C762T  Day 2  Day 4  C1410T (Day 2) | 7%  8%  8% | Yes  Yes  Yes |
| PB1 | G725T  Day 2  Day 4 | 100%  100% | Yes  YEs |
| PB2 | T318A  Day 2  Day 4 | 100%  100% | Yes  Yes |
| **A/H1N1, PA/I38T (London, ferret 360)** | | | |
| HA | G408T  Day 6  Day 8  Day 9  A511G  Day 6  Day 8  A697G  Day 6  Day 8  T698A  Day 6  Day 8  Day 9  C860T  Day 6  Day 8  C867T (Day 6)  G1366T  Day 6  Day 8  A1512C  Day 6  Day 8  Day 9 | 16%  21%  >95%  31%  17%  48%  5%  100%  100%  100%  25%  16%  10%  79%  28%  8%  48%  73% | Yes  Yes  Yes  No (K171E)  No (K171E)  No (K223V)  No (K223V)  No (I233K)  No (I233K)  No (I233K)  No (T287I)  No (T287I)  Yes  No (D456Y)  No (D456Y)  Yes  Yes  Yes |
| PA | C762T  Day 6  Day 8  Day 9  A1555G  Day 8  Day 9 | 10%  20%  20%  8%  53% | Yes  Yes  Yes  No (N519D)  No (N519D) |
| PB1 | G726T  Day 6  Day 8  Day 9  T1641C  Day 6  Day 8  C1392T  Day 8  Day 8  A2199G (Day 9) | 100%  100%  100%  19%  16%  48%  12%  22% | Yes  Yes  Yes  Yes  Yes  Yes  Yes  Yes |
| PB2 | G303A  Day 6  Day 8  Day 9  T318A  Day 6  Day 8  Day 9  C387A  Day 6  Day 8  Day 9  A690G  Day 6  Day 8  Day 9 | 14%  12%  32%  100%  100%  100%  16%  11%  23%  13%  18%  26% | Yes  Yes  Yes  Yes  Yes  Yes  Yes  Yes  Yes  Yes  Yes  Yes |
