# Supplementary material for: Evaluating the fitness of PA/I38T-substituted influenza A viruses with reduced baloxavir susceptibility in a competitive mixtures ferret model
Source: PLoS Pathog. 2021 May 6;17(5):e1009527. doi: 10.1371/journal.ppat.1009527 (PMC8130947; doi:10.1371/journal.ppat.1009527)
Supplement: S2 Table — (DOCX) [file ppat.1009527.s011.docx]

**S2 Table.** Amino acid and nucleotide difference in A/H1N1pdm09, A/H3N2, and type-B viruses isolated from baloxavir-treated patients.

|  | **Segment** | **A/H1N1pdm09** | | **A/H3N2** | | **B** |
| --- | --- | --- | --- | --- | --- | --- |
|  |  | **2HB001** | **2PQ003** | **344103** | **339111** | **286102** |
| **Amino acid** | HA | -* | - | - | - | - |
|  | NA | - | - | - | - | - |
|  | PA | I38T | I38T | I38T | I38T | I38T |
| **Nucleotide** | HA | - | - | T885C | - | - |
|  | NA | G1245A | - | - | - | - |
|  | PA | T113C | T113C | T113C | T113C  G1698A | T113C |

*No mutation was detected compared with the cognate wild type virus in amino acid or nucleotide.
