# Supplementary material for: Evaluating the fitness of PA/I38T-substituted influenza A viruses with reduced baloxavir susceptibility in a competitive mixtures ferret model
Source: PLoS Pathog. 2021 May 6;17(5):e1009527. doi: 10.1371/journal.ppat.1009527 (PMC8130947; doi:10.1371/journal.ppat.1009527)
Supplement: S1 Text — (DOCX) [file ppat.1009527.s012.docx]

**S1 Text. Supplementary methods**

- Cell culture
  - MDCK and MDCK-SIAT1 cells were obtained from the European Collection of Cell Cultures. MDCK cells were cultured in Minimum Essential Media (MEM) (Life Technologies) supplemented with 10% fetal bovine serum (FBS) (Sigma-Aldrich Co., LLC.) and 1% Kanamycin sulfate solution (Life Technologies). MDCK-SIAT1 cells were grown in Dulbecco’s modified Eagle’s medium (DMEM) (Sigma-Aldrich Co., LLC.) supplemented with 2 mM L-glutamine solution (Nacalai Tesque, Inc.), 1 µg/mL Geneticin^TM^ selective antibiotic (Thermo Fisher Scientific, Inc.), 10% FBS, and penicillin-streptomycin (Thermo Fisher Scientific, Inc.).
- Virus titration
  - The virus titer of reverse genetics-derived viruses was determined by the 50% tissue culture infectious dose (TCID_50_) method in MDCK cells in MEM containing 0.5% bovine serum albumin (BSA) (Sigma-Aldrich Co., LLC.), 1% Kanamycin sulfate solution, and 3 µg/mL trypsin (Sigma-Aldrich Co., LLC.). The virus titer of clinical isolates was determined by TCID_50_ method in MDCK-SIAT1 cells in DMEM containing 0.2% BSA, 25 mM HEPES (Thermo Fisher Scientific, Inc.), 2 mM L-glutamine solution, 1 µg/mL trypsin from bovine pancreas-TPCK treated (TPCK trypsin) solution (Sigma-Aldrich Co., LLC.), and penicillin-streptomycin.
- *In vitro* competitive fitness of rg virus pairs:
  - Competitive fitness experiments were conducted in MDCK cells using 50:50 mixtures of WT/I38X virus pairs prepared volumetrically based on infectious viral titers (TCID_50_). The presence of PA/I38X variant in cell culture supernatant was determined by Sanger sequencing.

Melbourne methods:

- Cell culture
  - MDCK-SIAT1 cells were obtained from ATCC. Cultures were grown in Dulbecco’s modified Eagle’s medium (DMEM) supplemented with 10% (v/v) fetal bovine serum, 2 mM GlutaMAX, 0.05% sodium bicarbonate, 100 μM MEM non-essential amino acid, 20 mM HEPES, 500 mg Geneticin and 50,000 U penicillin-streptomycin.
- Virus titration
  - The virus titer of clinical isolates was determined by the TCID_50_ method in MDCK-SIAT1 cells in DMEM containing 2 mM GlutaMAX, 0.05% sodium bicarbonate, 100 μM MEM non-essential amino acid, 20 mM HEPES, 100,000 U penicillin-streptomycin, 1000 μg Amphotericin B and 4 μg/mL of TPCK-treated trypsin.
- Ferret handling procedures
  - Identification/temperature-monitoring chips (LifeChip, Bio-Thermo) were implanted subcutaneously on the dorsal region of each ferret. Animals were monitored at least once daily for temperature and weight changes, had *ad libitum* access to pellet feed (Eukanuba) and water and were provided with supplementary wet food (Hill’s Pet Nutrition, Australia) as ethically required (below 90% baseline weight). Nasal wash samples were collected using 1 mL of PBS containing 1% w/v BSA. At the end of each experiment, ferrets were anesthetized before sacrifice by intracardiac injection of sodium pentobarbitone (≤1,000 mg/kg, Troy Laboratories).
- Pyrosequencing primers (Melbourne)
  - A/H3N2: Forward (Biotin-5’-TTGTCGAACTTGCAGAAAAGGC-3’), reverse (5’-GCCATTGTTCTGTCTCTCCCCT-3’) and pyrosequencing (5’-CATACCTCCAAGTGAGTGCA-3’, reverse orientation)
  - A/H1N1pmd09: Forward (Biotin-5’-CAATCCAATGATCGTCGAGC-3’), reverse (5’-GGTGCTTCAATAGTGCATTTGG-3’) and pyrosequencing (5’-CAAACTTCCAAATGTGTGCA-3’, reverse orientation)

London methods:

- Ferret handling
  - Body weight was measured daily, and strict procedures were followed to prevent aberrant cross-contamination between animals. Sentinel animals were handled before inoculated animals, and work surfaces and handlers’ gloves were decontaminated between animals.
- Cell culture:
  - MDCK cells were maintained in Dulbecco’s modified Eagle’s medium (DMEM; Gibco, Invitrogen) supplemented with 10% FBS and 1% penicillin/streptomycin (Sigma-Aldrich) and 1% non-essential amino acid (Gibco, Invitrogen).
- Plaque assays:
  - 100% confluent MDCK cell monolayers were inoculated with 100 μL of serially diluted samples and overlaid with 0.6% agarose (Oxoid) in supplemented DMEM with 1 μg trypsin (Worthington) mL−1 and incubated at 37°C for 3 days.
- Next-generation sequencing primer sets:
  - Full genome sequencing as described previously [1]
  - PA ORF amplified in two overlapping fragments PA-1 and PA-2. PA-1 forward (5’-AGCAAAAGCAGGTACTGATCCAA-3’), PA-1 reverse (5’- CTTGAATCAGTCAATTCACATGCC-3’), PA-2 forward (5’-ATGCAATCAAATGCATGAAGA-3’), PA-2 reverse (5’-ATATGGTCTCGTATTAGTAGAAACAAGGTACTT-3’)

**References**

1. Zhou B, Donnelly ME, Scholes DT, St George K, Hatta M, Kawaoka Y, et al. Single-reaction genomic amplification accelerates sequencing and vaccine production for classical and Swine origin human influenza a viruses. Journal of virology. 2009;83(19):10309-13. Epub 2009/07/17. doi: 10.1128/jvi.01109-09. PubMed PMID: 19605485; PubMed Central PMCID: PMCPMC2748056.
