# Supplementary material for: Evaluating the fitness of PA/I38T-substituted influenza A viruses with reduced baloxavir susceptibility in a competitive mixtures ferret model
Source: PLoS Pathog. 2021 May 6;17(5):e1009527. doi: 10.1371/journal.ppat.1009527 (PMC8130947; doi:10.1371/journal.ppat.1009527)
Supplement: Attachment — Submitted filename: Response to reviewers_I38T fitness 9 Mar 2021.docx [file ppat.1009527.s013.docx]

**Point by point response document to reviewer comments. All author responses are in *bold italics* and page numbers refer to the clean manuscript.**

**Part I - Summary**
Please use this section to discuss strengths/weaknesses of study, novelty/significance, general execution and scholarship.

Reviewer #3

Weaknesses:
1) Minor: Perhaps it would be convenient to show the study designs as another panel on the main text and not as supplementary material.

***We thank the reviewer for this comment. We have now included a new panel on Figure 1 (B) which outlines the competitive mixtures experiments for the in vitro experiments. In addition, we have included a new Figure 2 which covers the direct contact transmission chains and competitive mixtures experiments in ferrets from the Melbourne and London labs. We refer to this Figure throughout the results to aid a clearer understanding of the different experimental set-ups used.***

2) The ms would have been much stronger by including a group of ferrets prophylactically treated with baloxavir on days 1 or 2 prior to contact to better establish the relative fitness advantage of the PA I38T mutants.

***This is an important point and we thank the reviewer for raising it. The fitness experiments in ferrets were deliberately performed in the absence of baloxavir, in order to mimic a typical community scenario where the vast majority of infected person’s contacts would NOT be prophylactically treated. In other words, the most relevant fitness measurement of the PA/I38T variant is one that is calculated in the absence of drug pressure (as this is the reality in >99.999% of influenza patients). A possible exception to this may be in a nursing home setting, where symptomatic residents are treated with an antiviral while asymptomatic residents receive prophylaxis with the same antiviral.***

***Nonetheless, we have carefully considered the suggestion to evaluate the fitness of the PA/I38T virus in ferrets in the presence of baloxavir. Previous experiments in mice (Kuroda et al., ESWI 2020) suggest that the results of such a ferret study are likely to be quite predictable; baloxavir has greatly reduced inhibitory action on pure I38T viruses compared to WT viruses in vivo, therefore it would be expected that under drug pressure, the variant virus would be considerably fitter and rapidly outgrow the wildtype. As such, after discussion with our animal ethics committee, we instead chose to conduct these selection pressure experiments in cell culture, and are pleased to be able to now include these additional data in the manuscript. Competitive fitness experiments were conducted in the presence of four different baloxavir concentrations (1X, 10X, 100X and 1000X EC_50_) resulting in the selection of I38T-variant populations at different rates. These results allow the reader to understand the relative effect of the fitness of the variant /WT viruses with and without drug selective pressure (and with different levels of drug pressure). Text has been added to the manuscript in the results section (lines 166–182) and an additional figure in the supplementary section has been added (S2 Fig).***

**Part II – Major Issues: Key Experiments Required for Acceptance**
Please use this section to detail the key new experiments or modifications of existing experiments that should be absolutely required to validate study conclusions.

Reviewer #2:

1) Treatment emergent viruses were used in the transmission portions of some experiments, which is a strength of this manuscript. With a single in vivo exception (20:80, A/H3N2), I38 viruses clearly out-compete T38. But these studies are done in the absence of drug pressure. If even sub-effective concentrations were used in contacts or donors, what do the authors expect to see? These experiments should be piloted first in the primary airway cells to see if the competition patterns are sustained. If actual effective concentrations are used in vitro or in vivo, the data may look quite different, and at minimum should be introduced to temper the conclusions made in Lines 333-344.

***We thank the reviewer and agree that the manuscript would be strengthened by additional data in this regard. As mentioned in our response to a similar suggestion by Reviewer 3, our in vitro baloxavir sensitivity data combined with data from mouse models led us to infer that WT/I38T competition experiments in ferrets receiving baloxavir would result in a rapid selection of the I38T variant. We expect baloxavir inhibition of WT viruses to be dependent on the achievement of an effective plasma concentration of drug in treated ferrets, as has been reported in previous ferret studies (Lee et al., PLOS Path 2020).***

***However, to address the reviewer’s point we have performed competitive mixtures experiments in the presence of baloxavir in MDCK cells and the results have been included in the updated manuscript. As mentioned above, competitive fitness experiments taking place under baloxavir treatment selection pressure resulted in the fixation of I38T-variant populations by the third passage in MDCK cells (lines 166–182 and S2 Fig).***

2) Baloxavir marboxil is effective against influenza A and B viruses (and C and D), as the authors’ mention in the introduction. I38X substitutions can also arise and negatively affect B virus baloxavir susceptibility. The authors have not addressed the competitive fitness hypothesis with B viruses containing I38T. At minimum, this could have been done in the primary airway cells. While the they suggest in vitro influenza antiviral data is not always recapitulated in vivo/in circulation (297-299), this is not an acceptable answer to avoid such experiments in a basic science manuscript.

***Experiments examining our competitive fitness hypothesis with influenza B viruses were originally conducted in MucilAir cells alongside the influenza A viruses, but these data were omitted from the initial submission because we subsequently prioritised the testing of A/H1N1 and A/H3N2 isolates in ferrets. This prioritisation was due to (a) the limited number of experimental ferrets available and (b) the relative rarity of PA/I38T variants in influenza B viruses observed clinically (e.g. Hayden et al. NEJM 2018). However, we accept the reviewer’s critique and have therefore now included the influenza B in vitro data in the updated manuscript with appropriate descriptions in the results section (line 142–145, line 153 and Figure 1A).***

3) The manuscript would benefit from more discussion on the types of transmission event (contact vs indirect contact) that may or may not influence onward transmission of sensitive vs reduced susceptibility virus – citing and alluding to figure 6. It’s unfortunate that different proportions or the more ‘fit’ A/H3N2 I38T virus was not used with the London Study technique. Do the authors think either contact/fomite or airborne driven transmission could influence persistence and transmission of I38X viruses?

***This is a useful suggestion and we have now made additions to the discussion section which describe the different experimental setups, and how these may translate to real-life transmission settings (lines 346–348). We investigated direct vs indirect transmission routes using an A/H1N1 virus pair because the London laboratory had an established recombinant virus of this subtype, with well-characterised growth and transmission dynamics in ferrets using the experimental apparatus. We agree future studies could investigate the role of PA/I38T substitutions on transmission of A/H3N2 viruses in the indirect transmission model. Regarding potential differences in contact versus fomite transmission of I38X viruses, our experiments were unable to separate out these specific routes of transmission and to do so would require quite novel animal model designs. We have included discussion of these important points in the updated manuscript (line 359–371).***

**Part III – Minor Issues: Editorial and Data Presentation Modifications**
Please use this section for editorial suggestions as well as relatively minor modifications of existing data that would enhance clarity.

Reviewer #1: Major Comments:

-Authors mention in the introduction (line 111) and discussion (lines 316-318) that compensatory mutations can arise in viruses bearing variants associated with antiviral resistance. However, it appears that sequencing of ferret nasal wash specimens was limited to evaluation of the I38T residue alone. Was the rest of the PA/other segments sequenced as well to rule out compensatory mutations that may have arisen during virus replication, or to confirm that gain-of-function mutations were not acquired during serial passage transmission experiments?

***We thank the reviewer for this important suggestion. As a result, we have now completed whole-genome sequencing and included these data in a new results section (line 304–330), including an additional table in the supplementary materials (Supplemental Table 2) and text added to the discussion section (line 417–431). The sequencing was done for the Melbourne experiments using the pre-treatment (WT) and post-treatment (PA/I38T) clinical isolates, as well as the nasal wash samples at the peak of viral shedding in recipient ferrets (RF3s) where PA/I38T had become fixed. No non-synonymous mutations were detected that reached fixation alongside PA/I38T. In the pure I38T transmission chain (A/H3N2), a PB2/V89M substitution occurred at 68% frequency while in the 20% WT: 80% I38T group, a HA/T317I substitution was observed at 51% frequency. In addition, examination of the recombinant viruses by whole-genome sequencing revealed several non-synonymous mutations, including* *PB2/R508G (67% frequency), but these occurred in WT-dominated populations and are therefore not likely to result in compensatory fitness of PA/I38T viruses.***

-For in vitro experiments presented in Figure 1, the authors state (line 391) that supernatant was titered 48 hrs p.i. prior to passage, but it’s unclear if this was for the first passage only or every serial passage, please clarify.

***Text at line 502 in the methods section has been changed to clarify that supernatant was titrated at 48 h p.i. before passaging to fresh cells, and this was repeated with each subsequent passage. This was done to enable infection at 0.001 MOI for each passage.***

Furthermore, it would be helpful if the authors could present as supplemental (or state in the text) viral titer data captured during the in vitro serial passage experiments – especially in the case of the H1N1 wt vs I38T viruses, it would be helpful to see if virus supernatants containing higher levels of wt compared with I38T (from expts presented in 1B and 1C) maintained higher viral titers in vitro to support the somewhat higher titers in growth curves for wt virus presented in Figure 1A.

***We thank the reviewer for this insight and have included the requested data as supplementary Figures S7-9 and mentioned briefly in the text (line 501–502). For these in vitro and in vivo datasets, it is difficult to determine the correlation of infectious virus titer to the prevalence of WT or I38T-variant in the mixed virus populations, as the virus ratios we observe have arisen from competition – leading to the overrepresentation of WT-dominated populations fixed at near purity. A direct comparison between WT and I38T-variant titers in 100% infections found no significant differences in overall kinetics (Fig 3). As such, we do not observe substantial differences in viral titer depending on the relative prevalence of the I38T-variant in the competitive mixture population.***

For London in vivo experiments, were airborne transmission experiments performed with the wild-type and I38T viruses in pure populations prior to the competitive mixture experiments presented in Figure 6? It is currently unclear to the reader if the reduced airborne transmission fitness of the I38T virus on the H1N1 backbone relative to wild-type is because the wild-type virus outcompeted the I38T virus, or if the I38T virus did not exhibit robust transmissibility even in the absence of wild-type competition. The Melbourne data is strengthened by data presented in Figures S4 and S5 showing kinetics of pure population groups; having similar control data for the 1:1:1 transmission experiments would be of benefit.

***We thank the reviewer for this suggestion and we agree this is a limitation of the original study. Unfortunately, we are now not in a position to be able to complete the additional experiment due to resources of the London lab being currently at full capacity with essential SARS-CoV-2 work. As such, we have added a sentence to the discussion (line 362) which mentions that this is a limitation of the current study.***

-Figures 3, 5, and 6 report M gene copy numbers from ferret NW samples only, but the methods state that samples were titered for the presence of infectious virus in both experiments. Could this information be made available to the reader in supplemental form or otherwise?

***Graphs showing the infectious virus titers in Melbourne competitive mixtures experiments (S8 Fig.) and London experiment (S9 Fig.) have been added to the supplementary data.***

Minor comments:

-Please expand in the text (line 246) the first time ‘indirect transmission’ is used, to clarify that respiratory droplet/airborne route experiments are being conducted; the authors do specify this in the methods but it would be helpful to be explicit about this up-front, as ‘indirect transmission’ has also been used in the literature to describe transmission by environmental fomites.

***Text has been expanded at line 281–286 to define direct and indirect transmission as used in our studies.***

-Please provide reasoning why the H1N1 subtype was chosen for 1:1:1 transmission studies; was it because the Melbourne studies found that this virus exhibited enhanced within-host stability compared with the H3N2 virus subtype, or a different reason?

***The London group aimed to evaluate the PA/I38T substitution in a recombinant virus background, where competing viruses differed only by the amino acid change of interest. The recombinant A/England/195/2009 virus was selected as it had been previously used in the London laboratory in the direct/indirect contact 1:1:1 ferret transmission model, and therefore had well-characterised growth and transmission dynamics in ferrets. These experiments were planned and executed in parallel with the Melbourne studies, therefore the clinical H1 data had no influence on the planning of the London experiments.***

-Please specify culture temperature for primary human airway epithelial MucilAir cells experiments (lines 380-1)

***The cell culture conditions (37^o^C, 5% CO_2_) have been added to the text at line 492.***

-Please disclose in the text why H3N2 clinical isolate 253104 was not tested in Figure 1C.

***A 20:80 competition experiment was initiated with isolate pair 253104 in vitro. However, a procedural error led to the use of an incorrect infectious dose at passage 1, compromising the data for this experiment. The replication kinetics and competition trend in vitro for 253104 (Fig1A) is highly similar to the other two A/H3N2 virus pairs presented in fig 1, so its inclusion in the results does not significantly affect our interpretation of the in vitro data. Additionally, we did not use 253104 in our subsequent ferret studies. We have therefore removed the incomplete dataset for clinical isolate pair 253104 from the updated manuscript.***

Reviewer #2: 1) Line 52 – It is unclear what ‘remains low’ actually means. If more contemporary surveillance data, stats, percentages, is available to the authors’, this should be stated here and also in the introduction/discussion.

***We have amended the author summary at line 52 to state the percentage of virus isolates found to harbour known PA amino acid substitutions associated with reduced baloxavir susceptibility in global NIC/WHOCC surveillance data, as reported by Takashita et al. 2020 but also in the latest WHO reports. We have elaborated on this point in the introduction (line 109) and discussion (line 405).***

2) Could the authors please confirm in the materials and methods (Line 388) that the viruses were first diluted to equivalent TCID50 units/mL, and then mixed in in various volumetric ratios?

***The materials and methods have been clarified to state that viruses were first diluted to equivalent TCID_50_ units/mL, and then mixed at 50:50 or 20:80 volumetric ratios (line 499).***

3) I do not find the data and write-up for figure 4 to be particularly clear in the present version of the manuscript. From my perspective, some within-host/between-contacts statements are broadly derived from this figure, but may be more nuanced when taking into consideration specific aspects of figure 3 and 5. The authors’ may want to reconsider the portrayal and discussion of the data.

***We appreciate these data are complex and challenging to summarise in a single figure, but we do believe Figure 4 is the most appropriate way to bring all of the ferret data together in a visual manner. However, to try and draw out more of the nuances across Figures 3 and 5 (which are now Figure 4 and 6 in the revised manuscript) as suggested by the reviewer, we have indicated in the text the percentage change in I38T across the key experiments. We believe this addition, will allow the reader to have a clearer representation of the nuances of these data when digesting Figure 4.***

4) One unaddressed limitation is that different deep-sequencing protocols were used for each of the different centers or among the different in vitro and in vivo experiments, and lack of disclosure that variable protocols may miss minor populations that arise among the transmission events. In contrast, I’m actually less concerned with the ferret studies themselves being slightly variable among the centers – or being done at two different locations. Currently published baloxavir ferret data is largely in agreement, but given the costs and limited sample size associated with this model, the two-center approach is at least good insight, if not outwardly helpful to assess repeated measures. However, it's unclear why inter-center standardization of sample analyses wasn't applied.

***We acknowledge the lack of inter-centre standardisation is a limitation of the current study. With the limited time and resources available, the use of previously established laboratory protocols at each centre was the only feasible option. With regard to sequencing techniques, the Melbourne laboratory had the capacity to use a validated and previously published in-house pyrosequencing assay. Using this technique, we did not detect the emergence of alternate I38X substitutions in the Melbourne ferret experiments. However, pyrosequencing analyses are limited to short sequences targeted by the primers used, and therefore could not detect additional amino acid substitutions or nucleotide-level changes elsewhere in the genome which may have potentially arisen in the Melbourne experiment. We have added text to clarify the limitation of the variable sequencing protocols in the discussion (line 423).***

5) Line 289 – this statement doesn’t appear to be true for the 20:80 (I:T) mixture of A/H3N2

***This statement has been clarified to reflect that while the transmission chain initiated with a majority I38T variant population (20:80) resulted in the fixation of a pure variant population, the trend observed across multiple transmission chains – importantly, in both 50:50 groups – resulted in the fixation of the WT strain. Overall, this suggests the I38T substitution imposes a fitness disadvantage relative to WT in the tested H3N2 clinical isolate.***
